# Case Report: Successful Treatment of Cutaneous Squamous Cell Carcinoma in Three Patients With a Combination of Acitretin and Clarithromycin

**DOI:** 10.3389/fonc.2021.650974

**Published:** 2021-02-26

**Authors:** Yan Zhao, Yanting Zhu, Haiqing Wang, Chao Ji

**Affiliations:** Department of Dermatology, The First Affiliated Hospital of Fujian Medical University, Fuzhou, China

**Keywords:** cutaneous squamous cell carcinoma, acitretin, clarithromycin, combination therapy, complete regression

## Abstract

Few studies have evaluated acitretin as a primary treatment for cutaneous squamous cell carcinoma (CSCC). We report, for the first time, three cases of CSCC successfully treated with acitretin and clarithromycin. A literature review on this subject was also was performed. This case report included three patients with CSCC treated with acitretin and clarithromycin at the First Affiliated Hospital of Fujian Medical University (2008–2019). Patient 1 (83-year-old woman, ulcerated mass on the left cheek), patient 2 (97-year-old woman, painful mass on the left cheek) and patient 3 (76-year-old woman, large mass on the right ankle) received 8, 6, and 30 courses of combination therapy. All patients tolerated the adverse effects (pseudotumor cerebri and mucocutaneous dryness) and achieved complete regression within 6 months. Patients 1, 2, and 3 have not experienced recurrence during a 10-, 3-, and 6-year follow-up. Acitretin has limited efficacy as a monotherapy for CSCC. Our experience indicates that combination therapy with acitretin and clarithromycin may be an effective and well-tolerated treatment for unresectable CSCC.

## Introduction

Given its increasing incidence and potential for poor outcomes (1–3), cutaneous squamous cell carcinoma (CSCC) is emerging as a public health problem. High-risk CSCC can be difficult to treat by surgical excision because the neoplasm can extend or infiltrate beyond the visible borders of the lesion. A wide range of non-surgical treatments are available for high-risk CSCC. Acitretin is a second generation retinoid shown to reduce the incidence of new primary non-melanoma skin cancers in immunocompromised transplant recipients (4) and patients receiving BRAF inhibitor treatment (5). Acitretin is considered to have limited efficacy as a monotherapy for CSCC but seems more effective when used in combination with other agents (6, 7). Clarithromycin is an antibacterial drug that has been reported to enhance the anti-tumor activity of chemotherapeutic drugs *in vitro* and *in vivo* (8, 9). However, no previous study has evaluated the combination of acitretin and clarithromycin in the treatment of CSCC. We present 3 cases illustrating that combination therapy with acitretin and clarithromycin might be a promising option for patients with CSCC who are unwilling or unable to undergo surgery.

## Case Description

### Ethical Statement

The study was approved by the Medical Technology Clinical Application Ethics Committee of the First Affiliated Hospital of Fujian (No. [2015]084). Before treatment, the patients were fully informed about the potential advantages and disadvantages of combination therapy with acitretin and clarithromycin, including the possible side effects of these drugs. All patients provided written informed consent for treatment and agreed to attend follow-up visits every 2 weeks. All patients provided written informed consent for data analysis and publication.

### Patients

The present case report represents a retrospective analysis of the medical records of patients with CSCC who were treated with a combination of acitretin and clarithromycin at the Dermatology Department of the First Affiliated Hospital of Fujian between 2008 and 2018. Inclusion criteria consisted of a diagnosis of SCC by pathological biopsy and a history as well as tumor diameter ≥1 cm (10).

A total of 3 patients were involved in our study. Acitretin and clarithromycin were administered together to the patients from the time when skin biopsy confirmed the diagnosis of invasive SCC. We defined oral 30 mg/day of acitretin for 4 weeks and oral 500 mg/day of clarithromycin for 3 weeks as 1-month treatment course. There was no interval time between courses.

Once biochemical investigations, including measurements of serum creatinine, cholesterol, triglycerides, and potassium appeared abnormal, the treatment was stopped or the dosage was reduced.

### Patient 1

**Figure 1:**
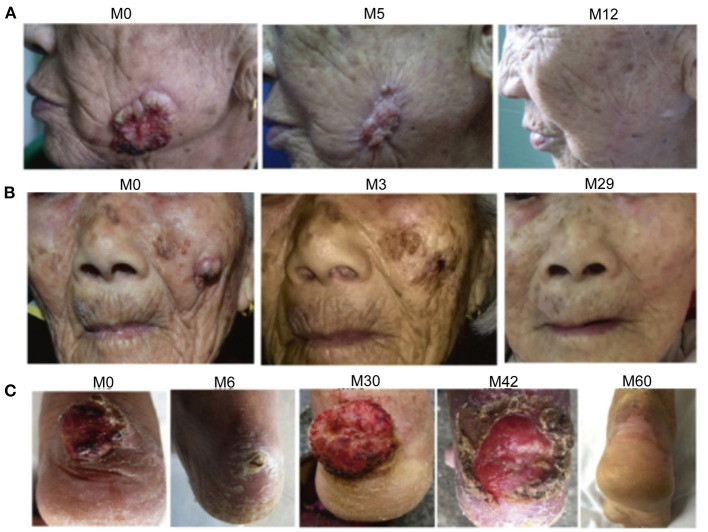
Photographs showing the regression of cutaneous squamous cell carcinoma (CSCC) in three patients treated with acitretin and clarithromycin. **(A)** Patient 1. The CSCC on the left cheek is shown at month 0 (before initiation of combination therapy), 5 and 12. **(B)** Patient 2. The CSCC on the left cheek is shown at month 0, 3, and 29. **(C)** Patient 3. The CSCC on the right ankle is shown at month 0, 6, 30, 42, and 60.

Patient 1 was an 83-year-old Chinese woman with a 2-year history of an asymptomatic ulcerated mass on her left cheek. Physical examination in March 2008 revealed a 4 × 3.5 cm crater-like mass with central ulceration on her left cheek (Figure 1A). Skin biopsy revealed the presence of atypical epithelial tumor cell formations extending beyond the epidermis into the underlying dermis, cornified cells and horny pearls, thereby confirming the diagnosis of invasive SCC. The patient showed a favorable response to acitretin and clarithromycin within 2 months after the first oral administration. The patient declined long-term treatment due to the development of adverse effects (pseudotumor cerebri and dryness of the lips and skin). Therefore, CO_2_ laser resection of the CSCC was performed after a total of 6 courses of combination therapy, and the patient then received an additional two courses of combination therapy. This patient has lived without tumor recurrence for 10 years.

### Patient 2

Patient 2 was a 97-year-old Chinese woman with a 1-year history of a painful mass on her left cheek. Physical examination in November 2015 demonstrated the presence of a 2 × 2 cm mass with an irregular edge and signs of erosion on her left cheek (Figure 1B). Skin biopsy confirmed the diagnosis of invasive SCC. The patient was treated with 6 courses of combination therapy. Darkening and regression of the tumor lesions were first detected in 2 months after the initiation of combination therapy, and a complete response was observed at 6 months. The only adverse effect reported by the patient was mucocutaneous dryness, but this resolved following cessation of treatment with acitretin. The patient has not experienced recurrence of CSCC during the 3-year follow-up.

### Patient 3

**Figure 2:**
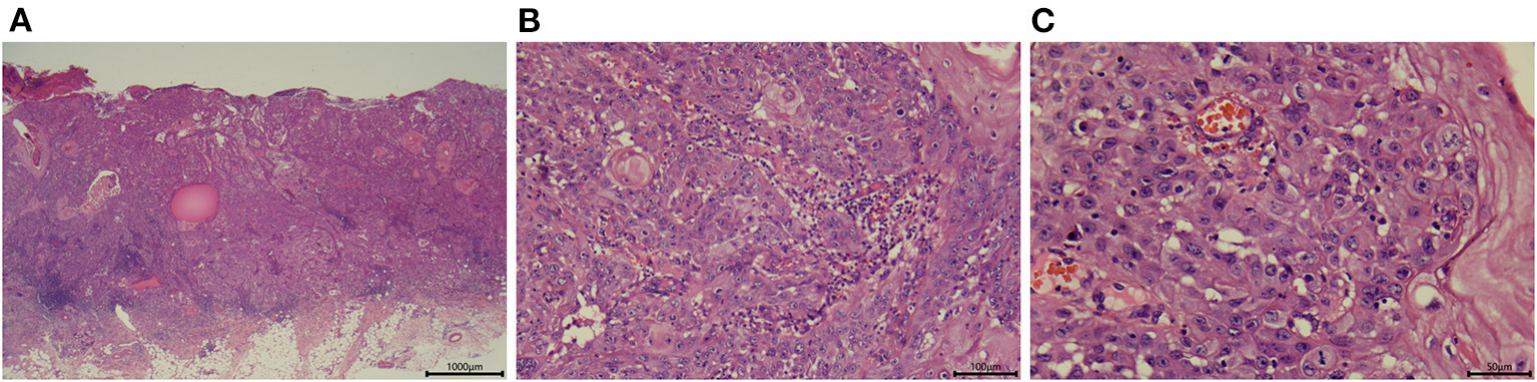
Representative images showing the histologic appearance of a tumor specimen from patient 3. **(A)** Section stained with hematoxylin-eosin (magnification, ×25). **(B)** Section stained with hematoxylin-eosin (magnification, ×200). **(C)** Section stained with hematoxylin-eosin (magnification, ×400).

**Figure 3:**
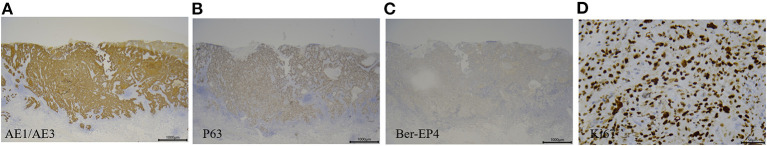
Immunohistology of tumor specimens from patient 3. **(A)** Tumor cells were positive for AE1/AE3 (magnification, ×25). **(B)** Tumor cells were positive for P63 (magnification, ×25). **(C)** Tumor cells were negative for Ber-EP4 (magnification, ×25). **(D)** Around 40% of tumor cells were positive for Ki67 (magnification, ×400).

Patient 3 was a 76-year-old Chinese woman with a 1-year history of a large mass on her right ankle that had progressively increased in size. Physical examination in September 2012 revealed a 4 × 4 cm mass with erosion on her right ankle (Figure 1C). Skin biopsy showed that the overlying epidermis was ulcerated with atypical keratinocytes invading the dermis. The keratinocytes were larger in the central parts of the tumor nests and formed eosinophilic horny pearls. The neoplastic keratinocytes had abundant eosinophilic and dyskeratotic cytoplasm and large vesicular nuclei with visible nucleoli and mitoses. An inflammatory reaction was evident, with many lymphocytes surrounding the stromal vessels and penetrating the tumor nests (Figure 2). Immunohistochemical analyses demonstrated that the tumor cells were positive for AE1/AE3 and P63, negative for Ber-EP4, and positive for Ki67 in about 40% of cells, confirming the diagnosis of invasive SCC (Figure 3). The patient was initially treated with 6 courses of combination therapy, which led to substantial regression of the tumor such that it resembled a small residual ulcer, and the patient then decided to discontinue the treatment. A local tumor relapse was detected 2 years later, so the patient resumed combination therapy and received an additional 24 courses of treatment, which resulted in complete remission. The only adverse effect experienced by the patient was mucocutaneous dryness. The patient remains alive and without tumor recurrence after a 6-year follow-up.

## Discussion

A notable finding of this case report was that combination therapy achieved CSCC regression in all three patients within 2–6 months after the first administered dose of acitretin and clarithromycin. Furthermore, two of the patients exhibited complete remission of the tumor without scaring, thereby avoiding the need for surgery, while the other patient was successfully treated by tumor excision after combination therapy. Importantly, all three patients remained free of recurrence during a follow-up of 3–10 years. In addition, all three patients tolerated the treatment and experienced only minimal adverse effects (pseudotumor cerebri and mucocutaneous dryness) that resolved after discontinuation of acitretin. To our knowledge, this is the first study to report that combination therapy with acitretin and clarithromycin may be an effective treatment for CSCC.

The majority of CSCCs can be successfully eradicated by surgical excision with post-operative margin assessment or microscopically controlled surgery, although the cosmetic results are not always satisfactory. When patients cannot or refuse to undergo surgery, alternative treatment options include radiotherapy, intralesional chemotherapy and photodynamic therapy. Radiotherapy is a potentially curative treatment in cases of early-stage CSCC where resection would cause substantial cosmetic or functional deficits. However, radiotherapy alone has limited effectiveness as a treatment for advanced CSCC and is associated with adverse effects. Anti-PD-1 antibodies are considered a first-line systemic treatment for patients with metastatic or locally advanced CSCC who are not candidates for curative surgery or radiotherapy, although its use in elderly patients is limited by the toxicity of this chemotherapeutic agent (11, 12). Recently, epidermal growth factor receptor inhibitors combined with chemotherapy or radiotherapy have been used as second-line systemic treatments for advanced CSCC (11, 13), but further research is needed to fully characterize the efficacy and safety of these strategies (11, 14). As awareness grows regarding the increasing number of cases of post-surgical CSSC relapse, it is becoming recognized that improved non-surgical treatments for CSCC are urgently needed.

Retinoids are established drugs and have been shown to reduce the development of non-melanoma skin cancer in high risk individuals. Previous randomized controlled trials have demonstrated that systemic retinoids can prevent new primary CSCC or new precancerous lesions in immunocompromised patients after transplantation (15). Typically, retinoids are considered for patients who develop multiple tumors each year, have multiple CSCCs in high-risk areas, or have numerous actinic keratoses. Acitretin is a second-generation retinoid reported to exhibit anti-tumor activity against actinic keratosis, verrucous carcinomas and SCC. Previous studies of the use of acitretin in the management of CSCC are rare and summarized in Table 1 (5–7). Two of these three studies were case reports describing the use of acitretin in the chemoprevention of CSCC in 8 patients receiving BRAF inhibitors (5) and one patient who had been treated with phototherapy (6). Only one prior study has described the use of acitretin to treat CSCC: the combination of acitretin, intralesional 5-fluorouracil and 5-fluorouracil chemowraps was found to cause complete regression of widespread CSCCs on the legs of two patients (7). To our knowledge, the present case report is the first to report the successful use of acitretin in combination with clarithromycin as a treatment for CSCC.

**Table 1:** Summary of publications reporting cases and our cases of cutaneous squamous cell carcinoma (CSCC) treated with acitretin.

**Reference**	**Patients**	**Treatment**	**Response**
Manalo et al. (7)	Two patients (74-year-old female and 60-year-old female) with widespread CSCCs on the legs	Acitretin (25 mg/d) combined with 5-fluorouracil and chemowraps for 6 months	Complete clinical resolution
Anforth et al. (5)	Eight patients treated with a BRAF inhibitor	Acitretin (10–15 mg/d) for 1–13 months	Suppression of CSCC development
Lebwohl et al. (6)	A 40-year-old male patient with psoriasis who developed CSCC following PUVA phototherapy	Acitretin (25 mg/d) for 25 months	Suppression of CSCC development
Patient 1	An 83-year-old Chinese woman with invasive SCC on the left cheek	Acitretin (30 mg/d) for 4 weeks and clarithromycin (500 mg/d) for 3 weeks in each course; 8 courses	Complete remission (6 months); 10 years without recurrence
Patient 2	A 97-year-old Chinese woman with invasive SCC on the left cheek	Acitretin (30 mg/d) for 4 weeks and clarithromycin (500 mg/d) for 3 weeks in each course; 6 courses	Complete remission (6 months); 3 years without recurrence
Patient 3	An 76-year-old Chinese woman with invasive SCC on the right ankle	Acitretin (30 mg/d) for 4 weeks and clarithromycin (500 mg/d) for 3 weeks in each course; 6 plus 24 courses resumed^*^	Complete remission (24 months resumed); 6 years without recurrence

* *After 6 courses of the combination therapy, this patient self-discontinued the treatment. Two years later, tumor recurrence was detected and the patient resumed the combination treatment for another 24 courses*.

Acitretin monotherapy is generally considered to have a low efficacy and is associated with dose-dependent side effects such as mucocutaneous dryness, hair loss, elevated triglycerides, transient elevations in liver function tests and (with long-term treatment) diffuse idiopathic skeletal hyperostosis. In view of the above issues, the patients in this case report were also administered clarithromycin, a macrolide antibiotic that has been reported to enhance the activity of conventional chemotherapy agents in cell lines and animal experiments (8, 9). Furthermore, long-term administration of clarithromycin was reported to prolong the survival time of patients with lung cancer (16). We speculate that the excellent responses to combination therapy observed in all three patients in the present report were due to the antiangiogenic and antitumor effects of clarithromycin, which synergized with the anti-tumor actions of acitretin.

Combination therapy with acitretin and clarithromycin was well-tolerated by all three patients in this study. We summarized the common characteristics of the three patients in our study which may somehow contributed to the successful treatment outcomes. We found that all of them were female older than 75 and refused large-scale surgical treatment. The pathological diagnoses were poorly to well-differentiated invasive SCC. Their tumors were all single and without any metastases. We believe that elderly patients with slow tumor growth respond well to drug treatment and this can help them to avoid the risks associated with surgery. For young patients with multiple or larger tumors, the drug treatment can also be used to reduce the size of the tumor, furthermore it will be beneficial to surgical resection. The only adverse effects reported were pseudotumor cerebri and mucocutaneous dryness, which are known side-effects of acitretin. Therefore, we suggest that the combination of acitretin and clarithromycin may represent an effective and safe therapy for CSCC.

As far as we are aware, this is the first study to report that combination therapy with acitretin and clarithromycin may be an effective and safe treatment for CSCC. Additional studies are required to further assess the efficacy and safety of combination therapy with acitretin and clarithromycin in the treatment of CSCC in larger populations and to investigate the underlying molecular mechanisms.

## Data Availability Statement

The original contributions generated in the study are included in the article/supplementary material, further inquiries can be directed to the corresponding author.

## Ethics Statement

The studies involving human participants were reviewed and approved by The study was approved by the Medical Technology Clinical Application Ethics Committee of the First Affiliated Hospital of Fujian (No. [2015]084). The patients/participants provided their written informed consent to participate in this study. Written informed consent was obtained from the individual(s) for the publication of any potentially identifiable images or data included in this article.

## Author Contributions

YZha: conception and design. CJ: administrative support. YZha, YZhu, and HW: provision of study materials or patients. CJ and YZhu: data analysis and interpretation. All authors: collection, assembly of data, manuscript writing, and final approval of manuscript.

## Conflict of Interest

The authors declare that the research was conducted in the absence of any commercial or financial relationships that could be construed as a potential conflict of interest.
